# The effect of inflammation and its reduction on brain plasticity in multiple sclerosis: MRI evidence

**DOI:** 10.1002/hbm.23184

**Published:** 2016-03-18

**Authors:** Valentina Tomassini, Alessandro d'Ambrosio, Nikolaos Petsas, Richard G. Wise, Emilia Sbardella, Marek Allen, Francesca Tona, Fulvia Fanelli, Catherine Foster, Marco Carnì, Antonio Gallo, Patrizia Pantano, Carlo Pozzilli

**Affiliations:** ^1^ Institute of Psychological Medicine and Clinical Neurosciences, Cardiff University School of Medicine United Kingdom; ^2^ Cardiff University Brain Research Imaging Centre (CUBRIC), Cardiff University School of Psychology United Kingdom; ^3^ IRCCS Fondazione Santa Lucia Rome Italy; ^4^ Department of Medical Surgical, Neurological, Metabolic and Aging Sciences, Second University of Naples Italy; ^5^ Department of Neurology and Psychiatry Sapienza University of Rome Italy; ^6^ IRCCS NeuroMed Pozzilli IS

**Keywords:** multiple sclerosis, functional MRI, inflammation, brain plasticity, interferon beta

## Abstract

Brain plasticity is the basis for systems‐level functional reorganization that promotes recovery in multiple sclerosis (MS). As inflammation interferes with plasticity, its pharmacological modulation may restore plasticity by promoting desired patterns of functional reorganization. Here, we tested the hypothesis that brain plasticity probed by a visuomotor adaptation task is impaired with MS inflammation and that pharmacological reduction of inflammation facilitates its restoration. MS patients were assessed twice before (sessions 1 and 2) and once after (session 3) the beginning of Interferon beta (IFN beta), using behavioural and structural MRI measures. During each session, 2 functional MRI runs of a visuomotor task, separated by 25‐minutes of task practice, were performed. Within‐session between‐run change in task‐related functional signal was our imaging marker of plasticity. During session 1, patients were compared with healthy controls. Comparison of patients' sessions 2 and 3 tested the effect of reduced inflammation on our imaging marker of plasticity. The proportion of patients with gadolinium‐enhancing lesions reduced significantly during IFN beta. In session 1, patients demonstrated a greater between‐run difference in functional MRI activity of secondary visual areas and cerebellum than controls. This abnormally large practice‐induced signal change in visual areas, and in functionally connected posterior parietal and motor cortices, was reduced in patients in session 3 compared with 2. Our results suggest that MS inflammation alters short‐term plasticity underlying motor practice. Reduction of inflammation with IFN beta is associated with a restoration of this plasticity, suggesting that modulation of inflammation may enhance recovery‐oriented strategies that rely on patients' brain plasticity. *Hum Brain Mapp 37:2431–2445, 2016*. © 2016 The Authors Human Brain Mapping Published by Wiley Periodicals, Inc.

**Abbreviations**
BOLD: Blood oxygen level dependent
CNS: Central Nervous System
EDSS: Expanded Disability Status Scale
FSL: FMRIB Software Library
T25‐FW: Timed 25 Foot Walk
FOV: Field of View
Gd: Gadolinium
GM: Grey matter
9‐HPT: 9 Hole Peg Test
IFN: Interferon
MNI: Montreal Neurological Institute
fMRI: functional MRI
MS: Multiple Sclerosis
PASAT: Paced Auditory Serial Addition Test
ROIs: Regions of interest
SE: Standard error
TE: Echo Time
TF: Thumb flexion
TR: Repetition Time
VBM: Voxel‐based morphometry
WI: Weighted image

## INTRODUCTION

The neural reorganization that mitigates the functional impact of damage [Enzinger and Fazekas, 2015] and promotes recovery in multiple sclerosis (MS) is based on brain plasticity [Tomassini et al., 2012a] that is the dynamic reorganization of brain function in response to experience (e.g., skill learning) [Doyon and Benali, 2005; Nudo 2013] or damage [Nudo, 2013]. Advanced neuroimaging shows that clinically meaningful functional changes occur during and after an active phase of MS [Lee et al., 2000; Mezzapesa et al., 2008; Pantano et al., 2002, 2005]. With this functional compensation, damage‐related altered patterns of functional reorganisation occur in MS, even early during the course of the disease [Pantano et al., 2002] or in the presence of preserved [Hulst et al., 2012] or fully recovered behaviour [Reddy et al., 2000]. These patterns of functional reorganisation differ from those of healthy volunteers even when recovery interventions drive performance improvements in the patients [Tomassini et al., 2012b]. While disability *per se* can sustain these abnormal patterns [Reddy et al., 2002], evidence suggests that MS inflammatory damage independently contributes to them [Lee et al., 2000; Reddy et al., 2002]. The effects of inflammation and its modulation on brain plasticity, however, remain largely unexplored, hampering the development of effective strategies enhancing recovery of function in MS patients [Tomassini et al., 2012a].

Indeed, experimental findings suggest that immune mediators, chronically over‐expressed in the MS inflammatory process, can affect the brain mechanisms underlying functional recovery by interfering with biochemical interactions, from a neuronal to a neurovascular level. Firstly, inflammation can influence the synaptic transmission, impairing the ability to encode and retain information [Di Filippo et al., 2014; Hauss‐Wegrzyniak et al., 2002; Min et al., 2009]. Electrophysiological studies support this mechanism [Mori et al., 2014; Nistico et al., 2013] and link it to clinically significant deficits [Mori et al., 2011]. Secondly, inflammation can damage connections between brain regions, hindering the spatial and temporal coherence of signal transmission [Allan and Rothwell, 2001; Di Filippo et al., 2008] necessary for plasticity to effectively drive recovery [Robertson and Murre, 1999]. Indeed, systems‐level plasticity relies on long‐range connections between brain regions. Damage to these connections occurs in MS [He et al., 2009; Petsas et al., 2013] and induces aberrant neural connectivity among widely distributed brain regions [Rocca et al., 2009]. Thirdly, inflammation can alter the neurovascular coupling [Girouard and Iadecola, 2006], a fundamental function of the brain that is the balance between energy demand imposed by neural activity and substrate delivery through blood flow, by interfering with the biochemical *milieu* that regulates the vasoactive chemicals supporting the normal neurovascular interactions.

As inflammation can interfere with brain plasticity in MS, its pharmacological modulation may restore plasticity by promoting desired patterns of brain functional reorganization. In this proof of concept study, we use advanced imaging methods to test whether brain plasticity is impaired as a result of MS inflammation and if a pharmacological reduction of inflammation through Interferon (IFN) beta may restore this plasticity. IFN beta reduces inflammatory activity in MS through a modulation of the pro‐inflammatory environment in the central nervous system (CNS) [Whitaker, 1994] that contributes to altered plasticity [Di Filippo et al.. 2014]. In patients with MS, the effects of IFN beta on MRI disease activity can be detected 4 weeks after the beginning of high dose IFN beta [De Stefano et al., 2012], suggesting that an early anti‐inflammatory effect can be expected in patients.

To probe plasticity and its changes over time, we used practice of a simple motor task that induces short‐term motor adaptation [Morgen et al., 2004], i.e., changes in sensorimotor cortical excitability and/or in the location or extent of cortical representations following repeated task execution that represent an optimized recruitment of the sensory‐motor network with practice [Butefisch et al., 2000; Classen et al., 1998; Hagenbeek et al., 2007]. The repetitive performance of simple and unskilled, but over‐learned movements can cause representational changes in MS patients that can be measured using functional MRI (fMRI) [Mancini et al., 2009]. Here, we hypothesised that training of these simple movements, requiring no fine hand coordination and very little strength, and inducing short‐term motor adaptation, caused brain functional changes that (i) were detectable in the MS patients, (ii) were altered as a result of inflammation and (iii) could be restored with immunomodulation.

## MATERIALS AND METHODS

### Participants and Study Design

**Figure 1:**
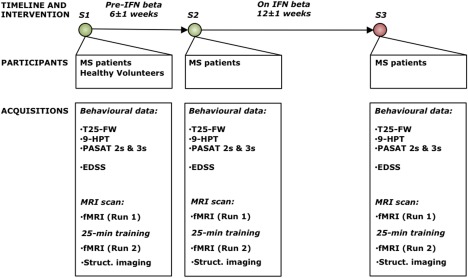
Study design and measurements. Patients were assessed using behavioural and MRI measures twice before (at session 1, week −6 ± 1, and at session 2, baseline) and once after (at session 3, week +12 ± 1) the beginning of IFN beta. Age‐ and sex‐matched healthy volunteers were assessed behaviourally and with MRI at session 1 only. During each session, participants were assessed using behavioural measures. Patients were also assessed using the EDSS score. Patients underwent conventional MRI acquisitions to detect the presence and quantify the number of Gd‐enhancing and T2‐hyperintense lesions, and to quantify GM volume. Motor task fMRI consisting of the repetition of directionally specific, voluntary, visually cued TF movements before (run 1) and after (run 2) 25 minutes of TF training was acquired in MS patients in the three scanning sessions and in healthy volunteers in session 1. The fMRI data analysis tested (**a**) within‐ and between‐group changes in brain activation associated with training at session 1; (**b**) changes in training‐related functional responses with IFN beta in the patients. *Abbreviations*: EDSS= Expanded Disability Status Scale; fMRI= functional MRI; Gd= Gadolinium; IFN= Interferon; GM= Grey Matter; 9‐HPT= Nine Hole Peg Test; PASAT= Paced Auditory Serial Addition Test; S= Session; T25‐FW= Timed 25‐Foot Walk; TF= Thumb Flexion; WI= Weighted Image.

Right‐handed patients with a diagnosis of MS according to the revised McDonald Criteria [Polman et al., 2005] and eligible to start treatment with IFN beta were recruited at Sapienza University of Rome. Steroid administration and relapse within 3 months of study entry were exclusion criteria. Patients were assessed using behavioural and MRI measures twice before (at session 1, week −6 ± 1, and at session 2, baseline) and once after (at session 3, week +12 ± 1) the beginning of IFN beta 1a 44 mcg (Rebif®, Merck Serono) subcutaneously administered 3 times weekly. The interval between sessions was chosen in order to balance the need to avoid a delay in treatment initiation for eligible patients, considered unethical, with the necessity to allow for a sufficient period of time for the drug to manifest an effect on MRI activity [De Stefano et al., 2012; Pozzilli et al., 1996]. Age‐ and sex‐matched healthy volunteers were assessed behaviourally and with MRI at session 1 only. Figure 1 reports the details of the study design and measurements.

Participants' written informed consent was obtained according to the Declaration of Helsinki and the protocol approved by the local Ethics Committee.

### Behavioural Measures and Statistical Analysis

During each session, participants were assessed using the Nine Hole Peg Test (9‐HPT), the Timed 25‐Foot Walk (T25‐FW) and the Paced Auditory Serial Addition Test (PASAT) 2s and 3s that are part of the MS Functional Composite (Cutter, Brain 1999). Patients were also assessed using the Expanded Disability Status Scale (EDSS) score [Kurtzke, 1983].

We used two‐tailed unpaired *t*‐test to investigate between‐group differences in age and behavioural measures. We used chi‐square test to assess sex differences between groups. To investigate changes in behavioural measures across sessions in patients we used repeated measures ANOVA followed by two‐tailed paired *t*‐tests that identified changes between pairs of sessions. For all the statistical tests, differences were considered significant at *P* ≤ 0.05. Values are reported as mean ± standard error (SE), unless stated otherwise.

### MRI Acquisitions and Analysis

Structural and functional MRI measurements were acquired using a Siemens Magnetom Verio 3T/70cm bore magnet (Siemens healthcare, Siemens, Germany). Analysis was carried out using tools from the FMRIB Software Library (FSL) (http://www.fmrib.ox.ac.uk/fsl) [Smith et al., 2004] and software for lesion volume quantification (Jim 5.0 software, Xinapse System, Leicester, UK; http://www.xinapse.com). All brain functional activations were labelled using the Montreal Neurological Institute (MNI) Structural Atlas, Harvard‐Oxford Structural Atlas, the Juelich Histological Atlas, the Cerebellar Atlas in MNI and the Oxford Thalamic Connectivity Probability Atlas (http://www.fmrib.ox.ac.uk/fsl/data/atlas-descriptions.html).

### Structural MRI

Patients underwent conventional MRI acquisitions that included T1‐weighted images (WIs) [Repetition Time (TR) = 550 ms; Echo Time (TE) = 9.8 ms; Field of View (FOV) =240 mm; matrix = 320 × 320; 25 axial slices, 4 mm thickness; 30% gap], acquired 5 minutes after the administration of Gadolinium (Gd) to detect the presence and quantify the number of Gd‐enhancing lesions; proton density and T2‐WIs (TR= 3,320 ms; TE1= 10 ms; TE2= 103 ms; FOV= 220 mm; matrix= 384 × 384; 25 axial slices; 4 mm thickness; 30% gap) to quantify T2‐hyperintense lesions; high resolution 3D T1‐WIs (2 acquisitions of 1 mm voxel size MPRAGE sequence with TR= 1,900 ms; TE= 2.93 ms; flip angle= 9°; FOV= 260 mm; matrix= 256 × 256; 176 sagittal slices; 1 mm thickness; no gap) to quantify grey matter (GM) volume. Healthy volunteers underwent 3D T1‐WIs only at session 1.

A trained researcher (F.T.) assessed the presence of Gd‐enhancing lesions on the post‐contrast T1‐WIs and calculated the volume of T2‐hyperintense lesions on T2‐WIs using a semiautomated technique (Jim 5.0 software, Xinapse System, Leicester, UK; http://www.xinapse.com). The statistical significance of the difference in the proportion of Gd‐positive *vs*. Gd‐negative scans was calculated using a non‐parametric binomial test with a significance level of *P* ≤ 0.05. 3D T1‐WIs were analyzed with FSL‐Voxel‐Based Morphometry (VBM) [Ashburner and Friston, 2000; Good et al., 2001], incorporating non‐linear registration and correction for local expansion or contraction to produce the GM template that was used as a covariate in the fMRI analysis to control for local differences in GM volume between patients and controls at session 1.

### Functional MRI

Motor task fMRI was acquired in MS patients in the three scanning sessions and in healthy volunteers in session 1. Whole brain functional images with blood oxygen level dependent (BOLD) contrast were obtained using gradient‐echo echo‐planar imaging (TR/TE = 3,000/30ms, 64x64 matrix, 50 transverse interleaved 3 mm slices, FOV 192 mm, flip angle 89°). Fieldmaps were also generated (TR = 488ms, TE1 = 4.92ms, TE2 = 7.38ms, 3 mm voxel size, 36 3‐mm interleaved transverse slices, FOV 250 mm, 64 × 64 matrix, flip angle 60°) for each individual to aid registration.

The motor task consisted of the repetition of directionally specific, voluntary, visually cued thumb flexion (TF) movements before (run 1) and after (run 2) 25 minutes of TF training [Morgen et al., 2004]. Participants performed 30‐second blocks of TF alternated with 30‐second blocks of rest. A colour fixation cross, flashing between orange (TF) and white (thumb relaxing with return to resting position) every 0.5 seconds, was used as cue to obtain a rate of 1 Hz TF. A 3‐second instruction of either “Flexion” or “Rest” condition preceded each block. The flashing cross was present throughout both conditions. Task instructions and pacing were visually presented using a MRI‐compatible stimulus‐presentation system with the use of goggles (VisuaStim Digital system from Resonance Technology Inc, Northridge, California). A researcher monitored the execution of the task to ensure consistency throughout the experiment.

The fMRI data analysis tested (a) within‐ and between‐group changes in brain activation associated with training at session 1; (b) changes in training‐related functional responses with IFN beta in the patients. Analysis was carried out using FEAT of FSL. The direction of functional contrasts is indicated in the text as *vs*., e.g. run 1 *vs*. run 2 indicates run 1 ‐ run 2 and run 2 *vs*. run 1 indicates run 2 ‐ run 1.

First‐level analysis (*step1*) was conducted in each participant and in each run to identify the main effect of the TF task. Data pre‐processing included motion correction, brain extraction, spatial smoothing (Gaussian kernel of 5‐mm full width at half maximum), high‐pass temporal filter (100‐s cut off) and correction for field inhomogeneities through fieldmap‐based echo‐planar imaging unwarping. Non‐linear registration from high‐resolution T1 structural to MNI standard brain space was carried out. The time series was analyzed using a general linear model approach with local autocorrelation correction. The canonical gamma variate hemodynamic response function was used. One explanatory variable (along with the temporal derivative) specified the onset and duration of the task periods to identify the mean effect associated with the task for both run 1 and run 2. Mixed effects, within session, higher‐level (group) analyses were conducted in patients and in controls [Beckmann et al., 2003] to identify the main effect of the task for run 1 and for run 2. Group *Z* statistical images were thresholded using clusters determined by *Z* > 2.3 and a cluster‐extent corrected significance threshold of *P* = 0.05 [Forman et al., 1995; Friston et al., 1994; Worsley et al., 1992].

Fixed effects, within‐session, higher‐level (group) analyses (*step 2*) were conducted in patients and in controls [Beckmann et al., 2003] to identify within‐subject between‐run signal changes in functional responses. Within‐session between‐run percent signal changes in functional responses represented the practice‐related change in brain activity that was our imaging marker of training‐related brain plasticity [Morgen et al., 2004].

Mixed effects, within‐session, higher‐level (group) analyses (*step 3*) to investigate the mean between run signal changes in both groups and to compare groups were conducted for session 1 [Beckmann et al., 2003] with automatic outlier de‐weighting [Woolrich, 2008]. GM partial volume information based on the individual structural images was added to the model as a voxel‐dependent covariate. Group *Z* statistical images were thresholded using clusters determined by *Z* > 2.3 and a cluster‐extent corrected significance threshold of *P* = 0.05.

Mixed effects, between‐session, within‐group paired higher‐level (group) analysis (*step 4*) was carried out with automatic outlier de‐weighting to detect changes in between‐run fMRI signal differences between sessions 2 and 3 in the patients. Group *Z* statistical images were thresholded using clusters determined by *Z* > 2.3 and a cluster‐extent corrected significance threshold of *P* = 0.05. Between‐run (session 2 *vs*. session 3) percent signal change in functional responses for the trained movements was considered as representing brain plasticity modulated by IFN beta anti‐inflammatory effects, an assumption discussed further below.

Considering the longitudinal design of the study and the absence of a placebo patient group, it not being ethically acceptable to withhold IFN beta treatment from a group of eligible patients, we expected that between‐session differences in between‐run changes in functional activity could be related to both a drug effect and a time effect not caused by the drug, but reflecting the natural history of reduction in MRI detectable active inflammation [Ciccarelli et al., 1999]. Therefore, for the purposes of exploring these effects in our data, without statistical testing, in the Discussion, we plotted the results of the between‐run changes in functional response using the fMRI signal extracted from the functionally defined, anatomically constrained, regions of interest (ROIs) within the cortical areas of the session 2 *vs*. session 3 group analysis (from *step 4*). Anatomical constraints were applied by multiplying the masks of the significant cortical areas (motor, parietal, temporal and visual cortices) from the MNI Structural Atlas with the binarized functional maps, thresholded using clusters determined by *Z* > 2.3.

## RESULTS

### Demographic and Clinical Characteristics

A total of 26 MS patients and 22 healthy volunteers were recruited. The demographic and clinical characteristics of the patients and healthy volunteers are reported in Table 1. Of the 26 patients who entered the study and completed the first assessment, 24 underwent all the scanning sessions. Two patients dropped out: one, after session 1, due to pregnancy and the other, after session 2, due protocol violation. In the 24 patients who completed the study, the mean ± SE overall study duration was 128.7 ± 4.8 days, with 45.2 ± 2.0 days between sessions 1 and 2 and 84.5 ± 4.2 days between sessions 2 and 3. During the study, there was no significant change in the EDSS scores or in the mean T25‐FW. A significant improvement in the mean 9‐HPT was observed for the dominant hand (*F* = 9.0, *df* = 1.9, *P* = 0.001) between sessions 1 and 2 (17.7 ± 0.4 *vs*. 16.8 ± 0.4, *P* = 0.02), and for the non‐dominant hand (*F* = 3.6, *df* = 1.5, *P* = 0.05) between sessions 2 and 3 (18.5 ± 0.5 *vs*. 17.8 ± 0.5, *P* = 0.03). The PASAT 3s (*F* = 15.2, *df* = 1.9, *P* < 0.0001) showed a significant change between sessions 1 and 2 (43.1 ± 1.9 *vs*. 49.2 ± 1.9, *P* < 0.0001), while the PASAT 2s (*F* = 10.4, *df* = 1.8, *P* < 0.0001) significantly changed both between sessions 1 and 2 (32.4 ± 2.6 *vs*. 35.8 ± 2.3, *P* < 0.0001) and between sessions 2 and 3 (35.8 ± 2.3 *vs*. 38.9 ± 2.8, *P* < 0.0001). None of the patients received steroids or experienced onset of new symptoms or worsening of previously reported symptoms during the study period.

**Table 1:** Cohorts' characteristics

	Patients (n=26)	Controls (n=22)	*P*
Age	36.1 ± 1.4	33.5 ± 1.7	0.23
Sex (F/M)	21/5	16/6	0.51°
Disease duration (months)	21.7 ± 6.1	–	–
EDSS score (median, range)	1.5, 0–3.0	–	–
Mean 9‐HPT (Right)	17.8 ± 0.4	15.8 ± 0.4	0.002
Mean 9‐HPT (Left)	19.3 ± 0.7	17.1 ± 0.4	0.01
Mean T25‐FW	5.8 ± 0.3	5.3 ± 0.1	0.09
No. correct responses PASAT 3s	43.1 ± 1.9	49.1 ± 2.3	0.05
No. correct responses PASAT 2s	33.8 ± 2.1	40.7 ± 2.4	0.04
No. Gd+ MRI scans	14/26	–	–
T2 hyperintense lesion volume (mm^3^)	2863.6 ± 531.1	–	–

Chi‐square test.

Values are reported as mean ± SE, unless indicated otherwise. The 9‐HPT and T25‐FW are expressed as the mean of two trials. Significance is tested using two‐tailed unpaired t test, unless stated otherwise. Abbreviations: EDSS = Expanded Disability Status Scale; Gd+ MRI scans= scan with at least one gadolinium‐enhancing lesion; 9‐HPT= 9‐hole peg test; PASAT= Paced Auditory Serial Addition Test; SE = standard error; T25‐FW= timed 25‐foot walk.

### Structural MRI Results

The initial structural MRI characteristics of the 26 patients who entered the study are reported in Table 1. Fourteen out of 26 (54%) MS patients during session 1 and 11 out 25 (44%) during session 2 had MRI scans with at least one Gd‐enhancing lesion, with no significant difference in the number of Gd‐positive *vs*. Gd‐negative scans (*P* = 0.5). At session 3 (on IFN beta), the number of Gd‐active scans (3 out of 24) reduced by 72% compared to session 2, with a significant difference in the number of Gd‐positive *vs*. Gd‐negative scans (*P* = 0.001).

### Functional MRI Results

The fMRI analysis was performed on 26 patients at session 1 and on 24 patients for sessions 2 and 3 of the study. All participants performed the functional task and training appropriately and completed the experimental protocol. In session 1, during run 1 (step 1 of fMRI analysis), the TF task engaged a large bilateral cluster of regions including the primary motor, premotor, posterior parietal and occipital cortices, as well as the basal ganglia and the cerebellum in both groups (Table 2). This cluster of regions was more extended in patients than in controls, including the left primary somatosensory cortex and the right V3 area in patients (Table 2).

**Table 2:** Main effect of the thumb flexion task (run 1, session 1) in patients and in controls

	Patients (n=26)				Controls (n=22)			
	MNI Coordinates				MNI Coordinates			
L PMd	10.5	−24	−14	66	10.2	−58	−2	34
R PMd	6.5	58	2	34	5.1	56	4	34
L SMC	10.8	−34	−14	62	10.3	−4	−2	54
R SMC	6.5	6	4	54	5.1	6	0	54
L M1	11.0	−34	−20	52	10.6	−34	−20	52
R M1	6.5	42	−12	52	3.2	42	−10	52
L S1	10.8	−30	−34	48	–	–	–	–
L IPL	11.2	−52	−32	40	11.3	−58	−36	24
R IPL	6.7	58	−22	40	4.3	64	−26	24
L SPL	6.3	−36	−44	48	5.3	−34	−48	48
R SPL	5.5	38	−44	48	4.9	38	−52	48
L V3	6.1	−28	−92	−2	3.02	−32	−94	−10
R V3	5.5	30	−88	−2	–	–	–	–
L V5	6.8	−50	−66	−2	6.1	−50	−64	−2
R V5	6.0	56	−60	−2	5.6	52	−58	−2
L Putamen	7.7	−24	−4	2	7.14	−26	0	2
R Putamen	6.1	24	4	−2	5.6	24	4	−2
L Pallidum	9.1	−22	−8	2	7.4	−20	−4	2
R Pallidum	6.4	22	−6	2	5.6	22	−6	2
L Thalamus (Premotor)	7.2	−10	−22	2	7.64	−12	−20	0
R Thalamus (Prefrontal)	7.0	10	−20	0	5.7	10	−22	0
R Cerebellum (lobule VI)	7.8	6	−62	−18	9.97	22	−66	−26
R Cerebellum (lobule VIIIa)	8.0	16	−66	−54	6.71	18	−64	−54

Localization of clusters is in Montreal Neurological Institute (MNI) Standard Brain Space. *Z* score of the peak voxel is reported for each cluster showing the main effect of the thumb flexion task in patients and in controls (random effects, *Z* > 2.3, *P* < 0.05, corrected). Thalamic and cerebellar regions connected to specific cortical regions are reported in brackets.

Abbreviations: PMd = dorsal premotor cortex; SMC = supplementary motor cortex; M1 = primary motor cortex; S1 = primary somatosensory cortex; IPL = intraparietal lobule; SPL = superior parietal lobule; V = visual cortex; R = right; L = left.

**Figure 2:**
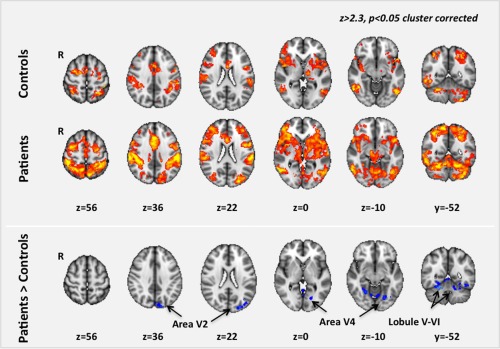
Training‐dependent fMRI signal changes in healthy volunteers and in MS patients during session 1. Maps of training‐related fMRI signal changes (contrasting run 1 *vs*. run 2) during session 1 are reported in healthy volunteers (indicated as controls) and in patients (*Z* > 2.3, *P* < 0.05, cluster corrected). Comparison between patients and controls showed a higher signal reduction in the patients in regions corresponding to the secondary visual areas (V2 and V4) and in the cerebellum (lobule V‐VI). *Abbreviations*: V= visual cortex; R= right hemisphere.

The within‐group between‐run (run 1 *vs*. run 2) changes in fMRI responses during session 1 (step 3 of fMRI analysis) are reported in Fig. 2 and in Table 3 for both patients and healthy controls. These changes involved a cluster of regions that included the prefrontal, premotor, primary sensorimotor, posterior somatosensory and visual cortices, as well as the basal ganglia and the cerebellum. During session 1, patients showed a greater between‐run (run 1 *vs*. run 2) signal reduction than controls in fMRI activity of secondary visual areas (V2 and V4) and cerebellum (lobule V‐VI) (Fig. 2 and Table 3).

**Table 3:** Training‐related functional changes

*Training‐related signal changes (run 1 vs. run 2) during session 1*								
	Patients (n=26)				Controls (n=22)			
	MNI Coordinates				MNI Coordinates			
L FP	4.4	−40	42	18	–	–	–	–
R FP	4.0	42	46	18	3.1	36	54	8
L IC	2.9	−38	2	4	3.2	−38	14	−4
R IC	3.9	36	2	4	3.1	32	14	8
L Cingulate	5.8	−6	10	34	3.5	−2	14	28
R Cingulate	5.2	6	14	34	3.0	8	10	36
L BA	4.7	−56	4	18	3.0	−54	2	18
R BA	4.0	56	10	26	3.6	52	4	26
L PMd	5.3	−26	−8	46	4.3	−52	2	36
R PMd	3.8	50	−2	46	3.7	44	2	46
L IPL	5.6	−56	−26	34	4.4	−58	−28	34
R IPL	5.2	60	−24	34	4.1	62	−22	34
L ITG	4.3	−48	−62	−14	4.2	−48	−58	−8
R ITG	5.6	54	−60	−14	4.9	56	−56	−8
L S1	5.2	−58	−22	36	4.0	−44	−24	36
R S1	3.8	58	−14	36	4.7	54	−20	36
L V5	5.5	−54	−70	4	4.2	−48	−72	−8
R V5	4.9	50	−60	4	2.9	54	−64	−8
L Caudate	2.9	−18	16	8	–	–	–	–
R Caudate	4.5	16	14	8	–	–	–	–
L Putamen	4.2	−26	−2	4	3.0	−20	10	0
R Putamen	2.9	30	4	6	2.7	22	14	−4
L Pallidum	3.7	−16	−2	0	2.9	−16	6	0
R Pallidum	3.0	20	0	0	2.9	20	4	0
L Thalamus (Prefrontal)	3.8	−14	−22	12	4.0	−12	−16	0
R Thalamus (Prefrontal)	4.8	10	−14	8	–	–	–	–
L Cerebellum (lobule VI)	5.4	−24	−56	−26	4.5	−28	−66	−24
R Cerebellum (lobule VI)	5.3	26	−52	−28	4.8	26	−52	−30
L Cerebellum (crus I)	5.4	−32	−68	−26	3.5	−38	−52	−36
R Cerebellum (crus I)	5.0	26	−72	−26	3.1	34	−56	−34
L Cerebellum (lobule VIIIa)	4.4	−16	−66	−50	5.0	−28	−58	−56
*Training‐related signal changes in sessions 2 and 3 in patients only*								
	Session 2 (n=24)				Session 3 (n=24)			
	MNI Coordinates				MNI Coordinates			
L FP	3.1	−40	38	22	3.8	−42	40	16
R FP	3.2	32	48	24	–	–	–	–
L IC	3.5	−32	16	4	4.4	−32	16	2
R IC	3.0	32	16	4	3.8	38	4	2
L Cingulate	2.9	−6	6	40	4.4	−8	16	28
R Cingulate	3.6	4	18	28	4.4	10	10	32
L PMv	3.7	−56	2	22	4.5	−58	4	24
R PMv	–	–	–	–	3.2	60	4	24
L S1	3.6	−42	−42	50	3.3	−40	−42	50
R S1	3.7	46	−34	52	2.8	38	−40	50
L IPL	5.6	−58	−32	34	5.5	−62	−28	34
R IPL	4.6	62	−28	34	5.0	64	−28	36
L V1	3.3	−10	−80	8	–	–	–	–
R V1	3.6	20	−74	8	–	–	–	–
L V2	3.3	−6	−86	−2	–	–	–	–
L V5	3.3	−50	−66	−2	2.9	−54	−64	−2
R V5	3.3	46	−64	8	–	–	–	–
R Caudate	3.6	16	14	6	–	–	–	–
L Putamen	–	–	–	–	3.8	−22	10	0
R Putamen	3.1	26	4	10	3.5	24	10	0
L Thalamus (prefrontal)	3.9	−10	−18	6	3.5	−8	−14	0
R Thalamus (prefrontal)	5.3	16	−12	6	4.6	10	−16	0
L Cerebellum (lobule VI)	3.5	−32	−52	−34	4.2	−30	−58	−32
R Cerebellum (lobule VI)	3.9	30	−52	−32	4.0	26	−64	−32
L Cerebellum (crus I)	4.9	−40	−52	−34	3.8	−40	−48	−34
R Cerebellum (crus I)	3.7	48	−54	−34	4.0	36	−52	−36
R Cerebellum (lobule VIIIa)	3.3	16	−64	−56	4.0	10	−64	−52

Localization of clusters is in Montreal Neurological Institute (MNI) Standard Brain Space. *Z* score of the peak voxel is reported for each cluster showing the effect of thumb flexion training in patients and in controls during session 1 and in patients only during session 2 *vs*. 3 (random effects, *Z* > 2.3, *P* < 0.05, corrected). Thalamic and cerebellar regions connected to specific cortical areas are reported in brackets.

Abbreviations: BA = Broca's area; FP = frontal pole; IC = insular cortex; IPL = inferior parietal lobule; ITG = inferior temporal gyrus; PMd = dorsal premotor cortex; PMv = ventral premotor cortex; M1 = primary motor cortex; S1 = primary somatosensory cortex; V = visual cortex; R = right; L = left.

There was an increase in fMRI responses during session 1 (run 2 *vs*. run 1) that involved the right angular gyrus and posterior cingulate gyrus bilaterally in the patients, and the right inferior parietal lobule and cingulate gyrus bilaterally in the healthy volunteers, with no significant between‐group difference (data not shown).

**Figure 3:**
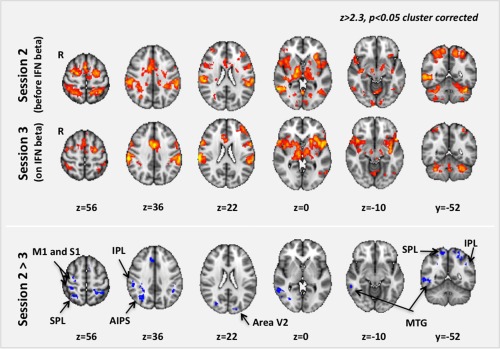
Training‐dependent fMRI signal changes in MS patients during session 2 *vs*. session 3. Maps of training‐related fMRI signal changes (contrasting run 1 *vs*. run 2) are reported in session 2 (before IFN beta) *vs*. session 3 (on IFN beta) in patients with MS (*Z* > 2.3, *P* < 0.05, cluster corrected). Comparing session 2 with session 3, patients showed a reduction of the training‐dependent fMRI signal changes after 12 weeks of IFN beta treatment in a cluster of regions encompassing the sensorimotor (M1, S1), temporal (MTG), visual (V2) and parietal (AIPS, IPL, SPL) cortices. *Abbreviations*. AIPS= anterior intraparietal sulcus; IPL= inferior parietal lobule; M1= primary motor cortex; MTG= middle temporal gyrus; S1= primary somatosensory cortex: SPL= superior parietal lobule.

Between‐run changes in fMRI activity in the patients for sessions 2 and 3 (step 4 of fMRI analysis) are reported in Fig. 3 and in Table 3. There was a reduction in the activation of cortical areas (run 1 *vs*. run 2) across sessions. Between sessions 2 (before IFN beta) and 3 (on IFN beta), patients showed a reduction of the between‐run signal change in the secondary visual areas (V2), as well, as in motor, temporal and parietal cortical areas (Fig. 3 and Table 3). There was no increase in the activation of cortical regions (run 1 *vs*. run 2) across sessions (session 3 *vs*. session 2).

## DISCUSSION

Our results suggest that MS inflammation alters plasticity underlying short‐term training of a simple motor task [Morgen et al., 2004], as reflected in abnormally greater practice‐related fMRI signal changes in the patients compared to healthy volunteers that were over and above structural differences in GM volume. Indeed, in session 1, patients showed a greater between‐run reduction than controls in fMRI activity of secondary visual areas and cerebellum (Fig. 2). This larger practice‐induced signal change in visual areas and in functional connected regions [Rizzolatti and Luppino, 2001] was reduced in the patients in session 3 compared with session 2 (Fig. 3), i.e., while on IFN beta, suggesting that a reduction of inflammation with IFN beta is accompanied by a restoration of systems‐level brain plasticity in the patients.

### MS inflammatory Activity on MRI and Its Changes With IFN Beta Treatment

Not all the MS patients who entered the study showed Gd‐enhancing lesions in their brains at the time of the baseline MRI scan. However, neuropathological evidence demonstrates that inflammatory activity typically spreads well beyond lesions visible on conventional MRI [Moll et al., 2011]. Indeed, conventional MRI is able to capture only part of the pathological processes underlying MS damage [Barkhof, 2002]. Also, Gd‐enhancing lesions represent a snapshot of inflammatory activity and thus their absence in the context of the natural history of the disease can reflect the dynamics associated with blood brain barrier alteration and repair [Tomassini and Palace, 2009].

While on IFN beta treatment, patients with Gd‐activity at session 1 showed a reduction in MRI disease activity. Evidence suggests that this reduction, observed after about 12 weeks of treatment, can already reflect the anti‐inflammatory effect of IFN beta [De Stefano et al., 2012; Pozzilli et al., 1996], thus offering a model to study the effect of rapid‐onset modulation of inflammation on brain plasticity.

### The Effect of the Motor Task and Training on Brain Functional Responses at Session 1

The motor task used for motor training consisted of the repetition of visually cued, directionally specific, simple thumb movements. As expected, this training led to changes in functional responses of a wide set of regions involved in visuomotor integration and motor execution both in patients and in controls [Morgen et al., 2004].

Training‐dependent reductions in brain activity were also different between groups and more pronounced in the patients in secondary visual areas (V2 and V4) and in cortical cerebellar regions connected to the primary and premotor cortices [O'Reilly et al., 2010]. This cluster of regions is involved in higher visuomotor control.

V2 and V4 are part of the ventral visual pathway that allows recognition of an object's characteristics for appropriate selection of motor plans [Fogassi et al., 2001; Schubotz and von Cramon, 2003]. V2 is involved in the storage of memory for object recognition and in the conversion of short‐term into longer‐term memories of objects [Lopez‐Aranda et al., 2009]. Although both V2 and V4 can be modulated by attention, V4 shows more pronounced changes in the spatial profile of its receptive fields with attention [Moran and Desimone, 1985] and thus is involved in encoding of stimulus salience. It is tuned for recognition of object features of intermediate complexity, like simple geometric shapes. Our task initially relied on external cues to drive the pace of thumb movements. As the participants practised the task, the need for attentional engagement when performing the visually cued movements will have decreased, while the representation of the cue pacing the task would have been stored as longer‐term memory of the object and incorporated in the internal sensorimotor model of the body‐environment interactions. The greater reduction in functional signal in visual regions in the patients could be explained by the greater effort spent in attending to the task at first exposure, before training, or, alternatively, by the more pronounced need to re‐build an internal model of body‐environment interactions with training on the task.

This functional difference in visual areas can be part of a long‐range modulation of higher motor control areas. Indeed, among the wide range of cortical connections, the cerebellum has connections with the visual cortices from lobules V and VI that represent primary sensorimotor zones [O'Reilly et al., 2010]. These zones contain overlapping functional connectivity maps for domain‐specific motor, somatosensory, visual and auditory cortices. The connections between cerebellar lobule V and VI and the extrastriate visual areas reflect the importance of visual information in motor control, as the cerebellum is essential to calibrate the relationship between visual and somatosensory/motor information. Indeed lobule V and VI are relevant for skill acquisition [Tomassini et al., 2011] and visuomotor adaptation [Della‐Maggiore et al., 2009]. The regions showing more pronounced reductions in functional responses with training in the patients were localised in lobule V and VI bilaterally, suggesting that the need for higher motor control based on external sensory feedback was greater at first exposure to the task or decreased more pronouncedly with practice, as an intrinsic modulation within the cerebellum set up accurately an acquired pattern of movements, possibly in concert with activation of motor‐related cortical regions [Halsband and Lange, 2006].

### The Effect of Pharmacological Modulation of Inflammation on Brain Plasticity

At session 1, patients showed a training‐related reduction in the functional signal of regions involved in higher visuomotor control that was more pronounced than the reduction observed in healthy volunteers. We interpreted these results as an expression of training‐related re‐building of an internal model of the body‐environment interactions in the patients that required progressively lower levels of motor control in order to set up accurately an acquired pattern of movements. Indeed, this larger practice‐induced signal change in visual areas and in functionally connected regions was reduced in the patients in session 3 compared with session 2. Specifically, we observed a reduction in training‐related signal changes in secondary visual areas and in connected posterior parietal and temporal regions. These regions encompass the ventral and dorsal streams that provide detailed representation of the visual world transformed into coordinates for skilled motor behaviour [Goodale and Milner, 1992]. Interestingly, between‐session changes in training‐dependent functional responses were observed also in the right primary sensorimotor cortices. The involvement of the right sensorimotor cortices (ipsilateral to the trained hand) has been associated with functional reorganisation and recovery in MS [Tomassini et al., 2012b]. Indeed, an increase in functional responses of the ipsilateral primary sensorimotor cortices has been associated with increasing levels of hand disability (Reddy H et al, Brain 2002), while a reduction in the functional signal of these same regions has been observed with training‐related improvements of hand movements [Tomassini et al., 2012a]. In this study, a reduction in functional changes between session 2 and 3 in these regions argues in favour of reduced involvement of the ipsilateral cortex in training‐related changes during pharmacological intervention and suggests that modulation of inflammation may influence the brain's functional connectivity at a systems‐level, possibly restoring patterns of normal activity [Pantano et al., 2002, 2005].

The influence of inflammation and its modulation on brain plasticity is expected on the basis of experimental evidence [Di Filippo et al., 2008]. Indeed, inflammatory molecules can interact with plasticity at a synaptic level [Di Filippo et al., 2014]. Many immune‐signalling molecules are expressed in the healthy brain and regulate synaptic function and plasticity [Pribiag and Stellwagen, 2014]. Also, neurons and glial cells can strictly interact [Perea et al., 2009]. The interaction between the immune and the nervous systems in the regulation of synaptic plasticity is needed for the physiologic development of neural circuitries [Wake et al., 2013] and for the regulation of plasticity and learning‐dependent synapse formation [Parkhurst et al., 2013]. However, during active neuroinflammation, microglial cells become activated and many immune molecules are released in higher concentrations with detrimental effects on the induction of brain plasticity. The brains of patients with MS are characterized by marked signs of immune cells activation/infiltration [Frohman et al. 2006], including activation of microglial cells, even in normal appearing white matter [van Horssen et al., 2012]. Thus, it is conceivable that the neuroinflammatory environment that characterizes MS may interfere with the neural circuits that subserve systems‐level plasticity. Experimental models of neuroinflammation support this immune‐neural interference and extend the concept of altered immune‐neural interactions to dysfunctional behaviour. The release of reactive oxygen species and pro‐inflammatory cytokines, along with the infiltration of immune cells and the activation of astrocytes and microglia, has been associated with abnormalities in synaptic plasticity [Di Filippo et al., 2014] and this altered synaptic transmission has been shown to parallel an impaired ability to encode and retain spatial information [Hauss‐Wegrzyniak et al., 2002; Kim do et al., 2012; Min et al., 2009], suggesting that inflammation‐related impairment in plasticity can also have behavioural consequences.

**Figure 4:**
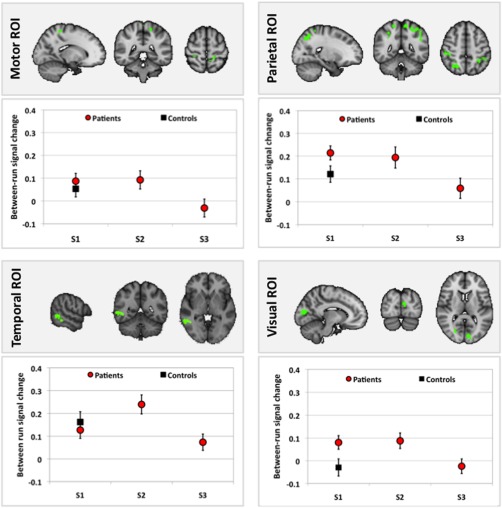
Variation in training‐induced fMRI signal changes over time in functionally defined regions of interest (ROIs). In the patients, differences across sessions in training‐related fMRI signal reductions (contrasting run 1 *vs*. run 2, with greater reductions shown as more positive values) are shown in ROIs derived from the map of the session 2 *vs*. session 3 contrast. In the healthy volunteers (indicated as controls) training‐related signal reductions are reported for session 1 only. In the patients, the training‐related reduction in functional signal was less in session 3 (on IFN beta) than in session 2 (before IFN beta) and largely stable from session 1 to session 2 (before IFN beta), suggesting a drug effect on brain plasticity. *Abbreviations*: ROI= region of interest; S= session.

Training‐dependent changes in functional responses between session 2 and session 3 paralleling the reduction in MRI disease activity with IFN beta suggest a drug‐related effect of modulated inflammation on plasticity (Fig. 4). The ROIs that are selected on the basis of a difference between sessions 2 and 3 are regions involved in motor training. These regions show little difference between sessions 1 and 2 and a more pronounced difference between sessions 2 and 3, with IFN beta treatment. Should between‐session changes reflect a session effect, they would be expected to be more pronounced during the first phase of the study, between sessions 1 and 2, which was also shorter than the intervention phase and thus more prone to repetition effects. Also, values from session 3 in the patients are similar to those of the controls suggesting a restoration of function in those regions that could result from reduction in inflammatory activity.

In this study, healthy volunteers were assessed only once, at session 1. A longitudinal assessment of functional changes in healthy controls was not included because the normal state of the brain did not allow us to explore changes in functional responses with changes in inflammation and we did not expect any biologically meaningful sustained effects of 25 minutes of motor training to carry over between imaging sessions 6 ‐ 12 weeks apart in the healthy controls.

### Limitations of the Study

We studied only one aspect of systems‐level brain plasticity that reflects the adaptation of functional responses with motor training. However, this type of plasticity is the basis of a resilient, yet adaptable behaviour and is probed in systems relevant for visuomotor integration, thus proving important and informative for rehabilitation [Bastian, 2008].

The time frame studied here is limited. While this restricts our ability to observe further and wider changes in training‐dependent plasticity with longer‐term modulation of inflammation, it allows us to pinpoint the anti‐inflammatory treatment effect without introducing the confounding factor of irreversible disease‐related structural changes that could affect the fMRI data interpretation.

The absence of an untrained group of patients makes it difficult to rule out completely the possibility that the difference between runs in each scanning session could be due to the passage of time rather than to effect of 25‐minute training. However, considering previous evidence on the successful induction of changes in functional responses using a similar adaptation task [Morgen et al., 2004], we assume that the training accounts for most of the between‐run difference in this study.

The patients included in the study had shown some clinical or sub‐clinical activity that made them eligible to start a disease modifying treatment, thus making the inclusion of a matched patient group acting as untreated control unethical. However, the lack of a patient group that could act as an untreated control makes it difficult to disentangle the treatment from the time effect, i.e., the effect of drug from the effect of a spontaneous reduction of active inflammation on the fMRI signal changes. One could argue that the observed changes in systems‐level plasticity are not necessarily related in all the patients to the anti‐inflammatory effects of IFN beta, which was used here to promote a rapid‐onset and consistent reduction in inflammatory activity in the patients [De Stefano et al., 2012] that paralleled training‐related changes in functional responses. Indeed, a reduction in inflammatory activity resulting from spontaneous evolution of MS lesions may have contributed to these functional changes [Tomassini and Palace, 2009]. However, should these changes have occurred as a result of natural history, they would still support our research hypothesis concerning the role of inflammation in altering plasticity.

The difficulty in interpreting the fMRI changes as being related to the effects of the drug derives from the potential session effect, i.e., the passage of time. However, the presence of 3 time points in the study design allows us to make predictions of signal changes related to the effects of drug or sessions. Here, we infer a drug effect on the assumption that changes associated with session effect on the functional signal would appear most strongly in the session 1 to session 2 differences, due to practice effects expected to occur more pronouncedly closer to the first exposure to the task and reducing with repeated exposure. We suggest, therefore, that the greater between‐run changes in fMRI activity for session 2 *vs*. 3 would be mainly due to the effect of reduced inflammation (Fig. 4). Under this reasoning, the different length of the two study phases would help to distinguish between session effect and the effect of modulated inflammation, i.e., the longer interval between session 2 and 3 made the functional changes less prone to session effect and thus more likely to highlight a drug effect. Therefore, we interpret the changes seen between sessions 2 and 3 as largely due to the effect of modulated inflammation, but we cannot completely exclude the potential influence of a residual session effect.

A specific interaction between drug‐induced immunomodulation and mechanisms of synaptic plasticity mediated by local modulation of cytokines and growth factors may have also contributed further to changes in systems‐level plasticity. Indeed, IFN beta has been shown to restore deficits of synaptic plasticity in MS patients [Mori et al., 2012] and this effect may be mediated by a modulation of the inflammatory *milieu*, with changes in the cytokine network that interfere with or favour processes of synaptic plasticity [del Rey et al., 2013; Khairova et al., 2009].

Finally, we cannot exclude that the changes during IFN beta treatment in brain functional responses could reflect a direct effect of IFN beta on mechanisms of brain plasticity. Although IFN beta only partially crosses the blood‐brain barrier, if directly administered on brain slices, it has the potential to modulate glutamate neurotransmission [Di Filippo et al., 2016], opening the possibility of a direct modulatory effect of the drug on plasticity, which is independent from its anti‐inflammatory action.

## CONCLUSION

Our results suggest that MS inflammation alters short‐term plasticity underlying motor training as reflected in greater practice‐related fMRI signal changes in the patients in the pre‐IFN beta phase when compared to healthy volunteers. Reduction of inflammation with IFN beta seems to restore this plasticity in the patients, at least within the range of damage and disability studied here, suggesting that modulation of MS inflammation can enhance recovery‐oriented strategies that rely on this plasticity.
